# Supplementary material for: The Fourth Dose of mRNA COVID-19 Vaccine Following 12 Different Three-Dose Regimens: Safety and Immunogenicity to Omicron BA.4/BA.5
Source: Vaccines (Basel). 2023 Mar 1;11(3):570. doi: 10.3390/vaccines11030570 (PMC10053821; doi:10.3390/vaccines11030570)
Supplement: Supplementary file 1 [file vaccines-11-00570-s001.zip › vaccines-2212540-supplementary.pdf]

Supplementary materials

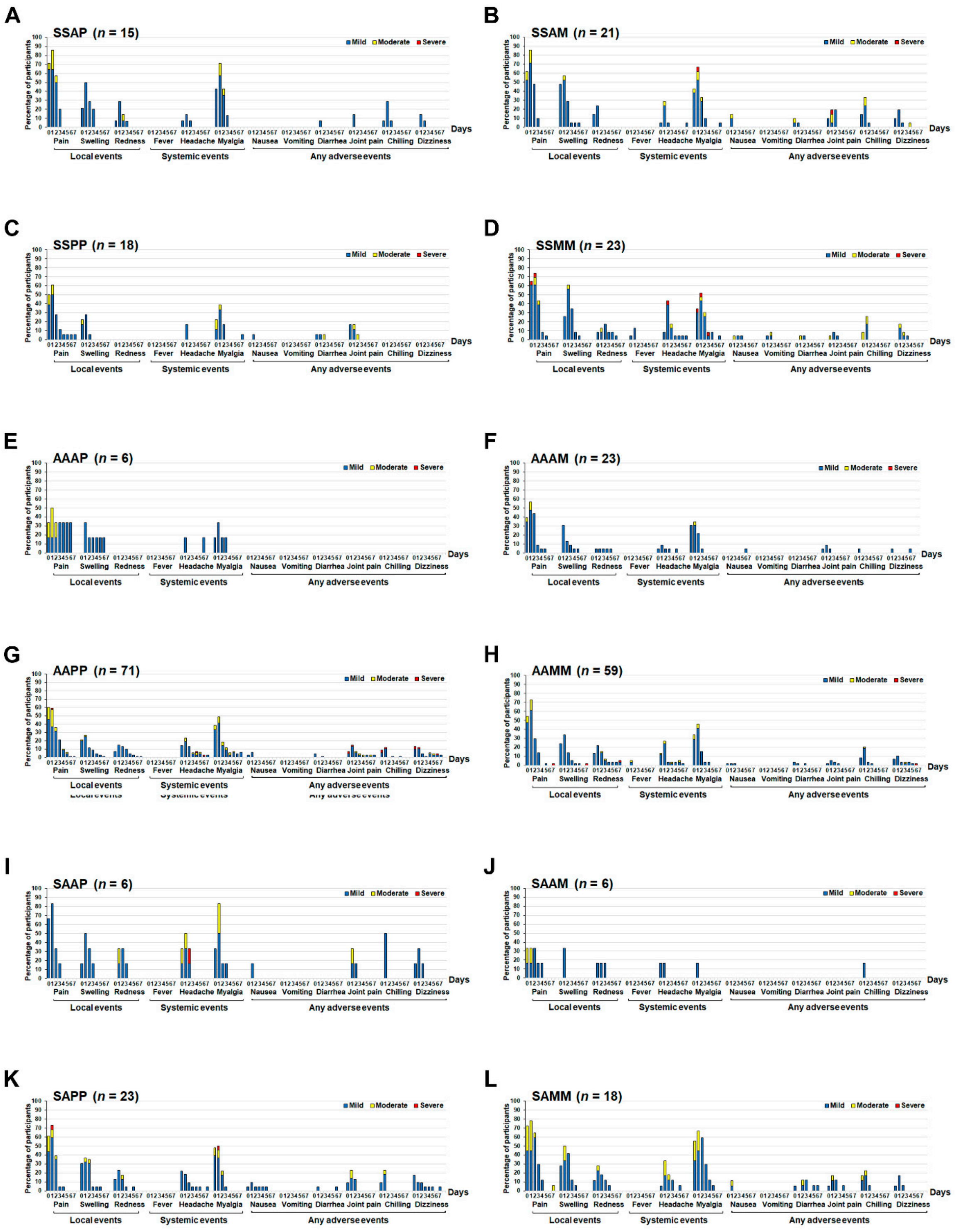

Figure S1. Solicited local, systemic, and adverse events of enrolled participants in different regimens. (A) SSAP, (B) SSAM, (C) SSPP, (D) SSMM, (E) AAAP, (F) AAAM, (G) AAPP, (H) AAMM, (I) SAAP, (J) SAAM, (K) SAPP, and (L) SAMM groups and the proportion of mild, moderate and severe adverse event 7 days after a booster dose for each group.
Abbreviations: A, AZD1222; M, mRNA-1273; P, BNT162b2; S, inactivated vaccine BBIBP-CorV or CoronaVac.

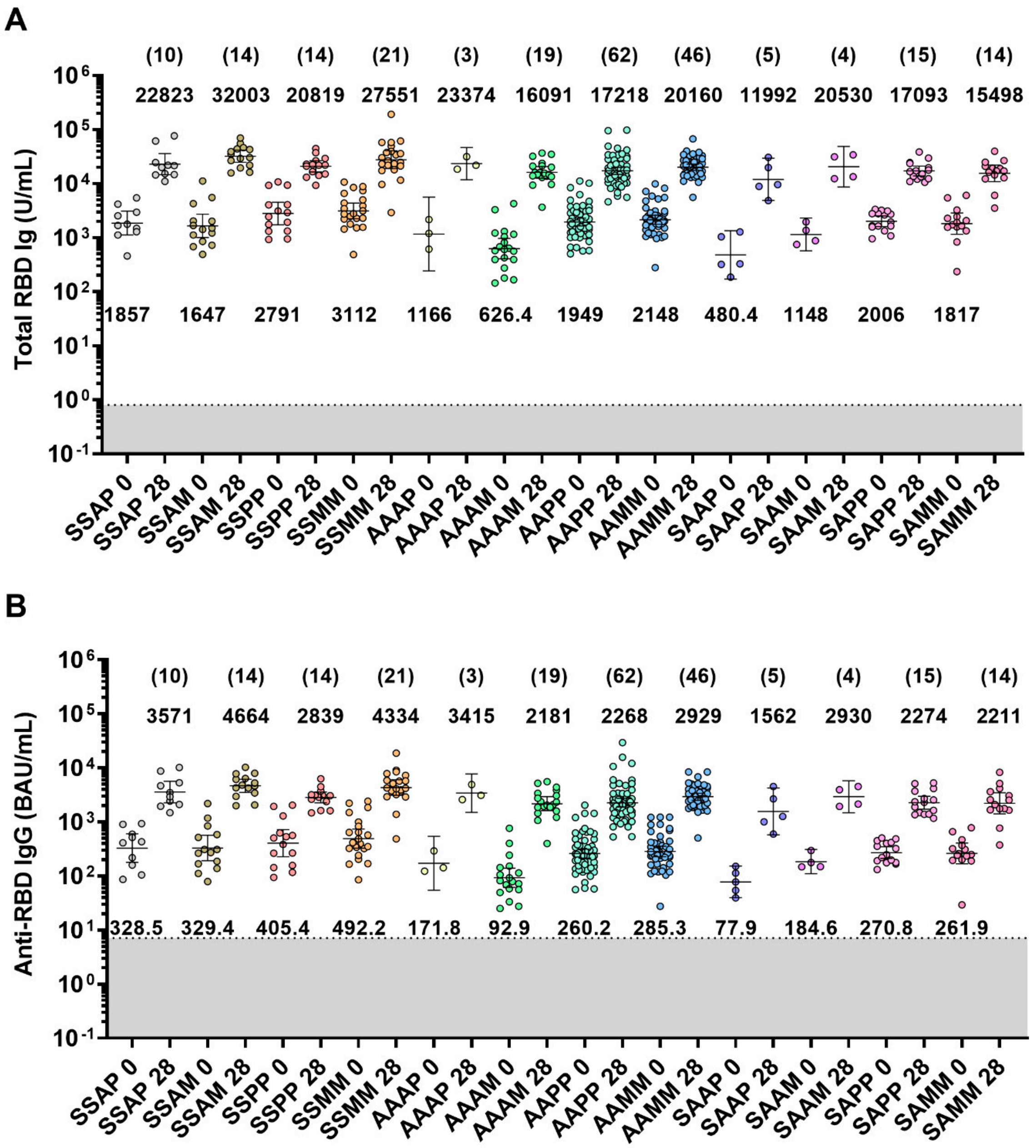

Figure S2. Binding antibody titers against the receptor binding domain of SARS-CoV-2 of the 12 different four-dose regimens. (A) Total immunoglobulin (Ig) specific for receptor binding domain (RBD) (Total RBD Ig) (U/mL) and (B) anti-RBD IgG (BAU/mL) were assessed for 12 different vaccine regimens on days 0 and 28 ± 7. The X-axis indicates the code of participant regimens, followed by the day of collection (0 and 28) The gray area indicates the seronegativity of total RBD Ig (<0.8 U/mL) or anti-RBG IgG (<7.1 BAU/mL). Each column shows a scatter plot with a geometric mean titer (GMT). The lines represent GMT with 95% confidence intervals (95% CI). Parentheses indicate the number of individuals in each regimen. Abbreviations: A, AZD1222; BAU, binding antibody unit; M, mRNA-1273; P, BNT162b2; S, inactivated vaccine BBIBP-CorV or CoronaVac.

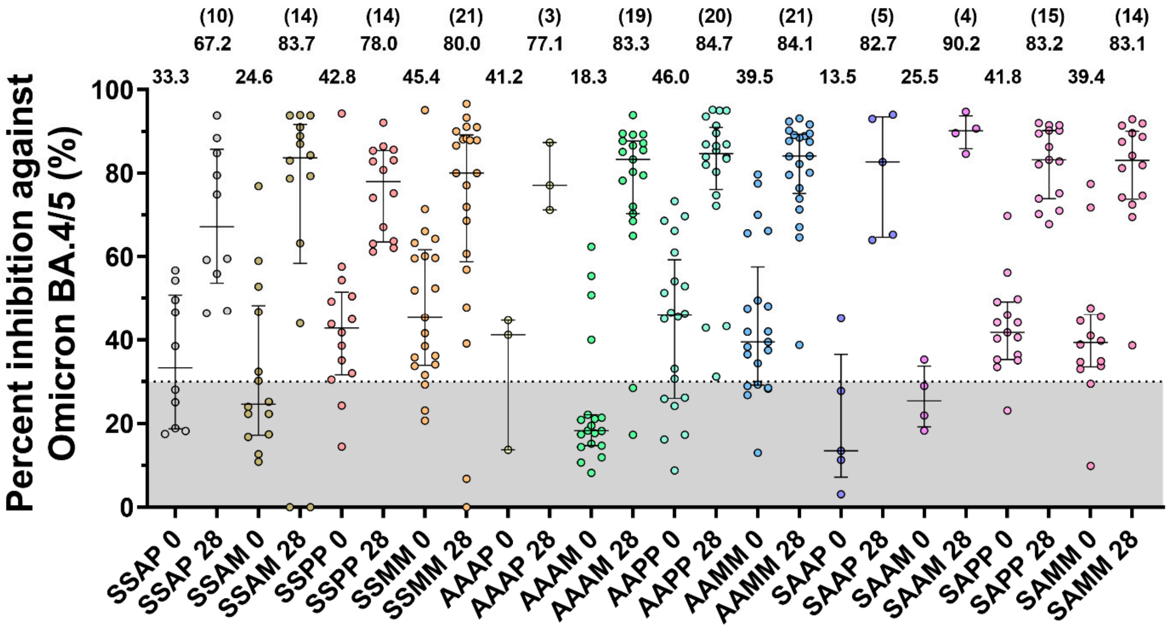

Figure S3. Neutralizing activity against the SARS-CoV-2 Omicron BA.4/5 of the 12 different four-dose regimens. Percent inhibition was assessed for 12 different vaccine regimens on days 0 and 28 ± 7. The X-axis indicates the code of participant regimens, followed by the day of collection (0 and 28). The gray area indicates the neutralizing activity of the SARS-CoV-2 Omicron BA.4/5 (<30%). Lines represent the medians (%) with interquartile range (IQR) values. Parentheses indicate the number of individuals in each regimen. Abbreviations: A, AZD1222; M, mRNA-1273; P, BNT162b2; S, inactivated vaccine BBIBP-CorV or CoronaVac.
